# Importin-11 is Essential for Normal Embryonic Development in Mice

**DOI:** 10.7150/ijms.40697

**Published:** 2020-03-12

**Authors:** Ju-Young Lee, Faiz Ur Rahman, Eun-Kyeung Kim, Sang-Mi Cho, Hae-rim Kim, Kihoon Lee, Chin-Soo Lee, Won-Kee Yoon, Ok-sung Moon, Young-won Seo, Young-Suk Won, Hyoung-Chin Kim, Bae-Hwan Kim, Ki-Hoan Nam

**Affiliations:** 1 Laboratory Animal Resource Center, Korea Research Institute of Bioscience and Biotechnology, Yeonjudanji-ro 30, Chungbuk 28116, Korea.; 2 Department of Public Health, College of Natural Science, Keimyung University, Daegu, 42601, Korea.

**Keywords:** Importin-11, embryonic lethal, null mutation, knockout, embryonic development, phenotype

## Abstract

Importin-11 (Ipo11) is a novel member of the human importin family of transport receptors (karyopherins), which are known to mediate the nucleocytoplasmic transport of protein and RNA cargos. Despite its role in the transport of protein, we found that knockout of Ipo11 nuclear import factor affects normal embryonic development and govern embryo-lethal phenotypes in mice. In this study, we for the first time produced a mouse line containing null mutation in *Ipo11* gene utilized by gene trapping. The *Ipo11^-/-^* embryos showed an embryonic lethal phenotype. The *Ipo11^-/-^* embryos showed a reduced size at embryonic day 10.5 (E10.5) when compared with *Ipo11^+/+^* or *Ipo11^+/-^* embryos and died by E11.5. Whereas *Ipo11^+/-^* mice were healthy and fertile, and there was no detectable changes in embryonic lethality and phenotype when reviewed. In the X-gal staining with the *Ipo11^-/-^* or *Ipo11^+/-^* embryos, strong X-gal staining positivity was detected systematically in the whole mount embryos at E10.5, although almost no X-gal positivity was detected at E9.5, indicating that the embryos die soon after the process of Ipo11 expression started. These results indicate that Ipo11 is essential for the normal embryonic development in mice.

## Introduction

To begin with, nuclear and cytoplasmic compartments are separated by nuclear envelope in eukaryotic cells. Through nuclear pore complexes (NPC), the nuclear envelope permits macromolecules to be exchanged between the two compartments 1. In this sense, the nucleocytoplasmic transport system functions as a key mediator of signal transduction by regulating protein localization. In fact, specific signal sequences called nuclear localization signals (NLSs) are required by nuclear import of proteins, and importin α/β heterodimer recognized the basic type of NLS and targeted to nuclear pores. More importantly, the importin α/β heterodimer targets hundreds of proteins to the NPC, and mediate their translocation across the nuclear envelope 2,3.

Broadly speaking, multiple regulatory mechanisms such as cell cycle, transcription, RNA processing, signal transduction and circadian rhythms, are also being identified that control cargo-carrier interactions and are regulated at the level of nucleocytoplasmic transport 4.

In this relation, karyopherins are a group of proteins including both importins and exportins, comprise a conserved family of mobile targeting receptors mediating transportation of molecules between the cytoplasm and the nucleus of a eukaryotic cell 5.

To begin with, importins are members of a family of transport receptors that mediate the nucleocytoplasmic transport of protein and RNA cargoes, exhibiting as a master regulator of nucleocytoplasmic transport 6, interacts with the Ran GTPase, and constitutively shuttles between the nuclear and cytoplasmic compartments. In other words, importin-11 a member of transport receptors mediates the nuclear import of UbcM2, a murine E2 ubiquitin-conjugating enzyme, exhibiting as a specific import receptor for UbcM2 7. Furthermore, importin-11 along with ubiquitin-conjugating enzyme regulates the localization and activity of the antioxidant transcription factor NF-E2 p45-related factor (NRF2) during homeostasis 8. Broadly speaking, importin-11 has also a distinct role in regulating ribosome synthesis and/or maturation by being a mediator of ribosomal protein L12 (rpL12) nuclear import 9. It is important to realize that importin‐11 plays roles in synaptic development and functions in* Drosophila.* In the case of importin‐11 mutant flies, characteristic defects can be seen in the synaptic transmission in adult photoreceptors and at larval neuromuscular junctions (NMJs). Furthermore, this acts as a characteristic factor resembling the phenotype of bone morphogenic protein (BMP) pathway disruption. Neurons deficient in importin-β11 were viable and properly differentiated, but showed distinct defects 10. Moreover, Wnt signaling pathways in *Drosophila* required importin-11 to adjust the synaptic development at NMJs 11. Besides this, overexpression of importin-11 can promote bladder cancer (BCa) progression, invasion and migration 12. Importantly, it has been also reported that altering the levels of nuclear import factors importin α in early *Xenopus laevis* embryos affects later development 13.

In the present study we analyzed that how deficiency of importin-11 in the mouse affects normal developmental processes utilizing *in vivo* approaches. Our results demonstrated that Ipo11*^-/-^* embryos died by embryonic day 11.5, and showed that the lethality is closely related with the start of embryonic expression of Ipo11. Our data suggested that importin-11 knockout mice showed embryo-lethal phenotypes.

## Materials and Methods

### Generation of the Ipo11-deficient mouse

It is noted that for the generation of Ipo11 knockout mice, a mutation of *Ipo11* gene was introduced into KTPU8 ES cells by insertion of pU-21T gene trap vector with electroporation as described previously 14,15. Overall, the trap vector contains a splice acceptor site, a cDNA encoding the β-galactosidase reporter linked to neomycin (β-geo), and a polyadenylation site. After the neomycin selection, the vector insertion in the ES cell clones were identified by the presence of vector specific DNA sequences with PCR primers; lox71-p (5'-GGTCGAGGGACCTATACCGTT) and SA-9 (5'-AGAAATTGATGATCTATTAA) for 5' region and pupa-S (5'-AGAAATTGATGATCTATTAA) and T7 (5'-CCCTATAGTGAGTCGTATTA) for 3' region of the vector. Notably, the exact vector insertion site on the *Ipo11* gene was determined by inverse PCR and nucleotide sequencing as described previously 14. Accordingly, the PCR primers used for identification of mutant allele were a mutant specific forward and reverse primer pair; 5'- GCTGTGATCCTTGTGGAATACT-3', and 5'-CAAACCCAAAAGGGTCTTTGAG-3', and a wild specific forward and reverse primer pair; 5'-GCTGTGATCCTTGTGGAATACT-3' and 5'- CAAACCCAAAAGGGTCTTTGAG -3'. In this case, the PCR product sizes for wild and mutant alleles were 514 and 322 base pairs, respectively. The ES cells from a selected mutant ES cell clone were microinjected into blastocysts from C57BL/6J. Next, these blastocysts were subsequently transferred into pseudo-pregnant mice to obtain chimeric mice. These chimeric male mice were mated with albino C57BL/6J females to establish a germ line transmission of mutant allele. These characterized germline transmitted mutant mice were next backcrossed with C57BL/6J for at least for six generations before experiments. All animal experiments were conducted with the approval of the ethics committee of the Korea Research Institute of Bioscience and Biotechnology (KRIBB). All animals were bred and maintained in a SPF facility under a 12 h light-dark cycle (lights on at 7:00 and light off at 19:00) at 22 ± 0.5 °C and humidity of 55 ± 15%. They were frequently provided free access to food and water. All the procedures used in this study were performed in accordance with our institutional guidelines and regulations.

### RT-PCR analysis

To see the Ipo11 gene expression levels in the embryo, the total RNA was extracted from whole embryos at embryonic day 10.5 (E10.5) using the total RNA and protein isolation kit (MACHEREY-NAGEL, Inc., Duren, Germany) according to the manufacturer's instructions. The RNA concentration was determined with Eppendorf BioPhotometer (Eppendorf AG, Hamburg, Germany). The cDNA synthesis was performed with the Superscript IV Reverse-transcriptase System (Invitrogen, Carlsbad, CA) according to the manufacturer's instructions. Briefly, the cDNA product was amplified by PCR using the primers specific for exon 22 and 30 of *Ipo11* gene (a forward primer 5'-CACACCAGAGCTGCTTCGTA-3' and a reverse primer 5'-TTTCCATGAGGGACTGGAAG-3'), with the following reaction conditions: 94 °C for 5 min, followed by 30 cycles of 94 °C for 1 min, 58 °C for 2 min, and 72 °C for 1 min, and a final extension at 72 °C for 5 min. β-actin (forward primer : 5'-ATCGTGGGCCGCCCTAGGCACC-3' and revers primer : 5'-CTCTTTAAGTCACGCACGATTTC-3') was used as an internal control.

### Western blot analysis

Specifically, the protein was extracted from the whole embryo at E10.5 using a lysis buffer (150 mM NaCl, 50 mM Tris-HCl (pH 8.0), 1% NP-40, 5 mM EDTA, and 1 mM PMSF). The extracted protein was separated on 8% sodium dodesylsulfate-polyacrylamide gel electrophoresis (SDS-PAGE), and transferred onto PVDF membrane (MILLIPORE, Damstadt, Germany). In this event, the membrane was blocked with 5% skim milk in TBST (50 mM Tris-HCl, pH 7.4, 150 mM NaCl and 0.1% Tween20). Additionally, the membranes were probed with an anti-Ipo11 antibody (dilution 1:1000, Proteintech, USA) overnight at 4℃, and then treated with the horseradish peroxidase conjugated secondary antibody in TBST. At that time, the proteins were detected with the enhanced chemiluminescent (ECL) protein detection system (Millipore). Chiefly, the glyceraldehyde 3-phosphate dehydrogenase (GAPDH) (dilution 1:5000) was used as an internal loading control.

### X-gal staining of mouse embryos

For whole-mount X-gal staining, the embryos were fixed with a fixation buffer (1% formaldehyde, 2% glutaraldehyde, 0.02% NP-40, in 1× PBS) at 4 °C for 2 hours. Then they were washed with a washing solution (1M MgCl2, 10% NP-40, 5% Na-Deoxycholate, in 1× PBS) for 20 min twice. The embryos were then incubated with X-gal staining solution (5 mM K3Fe (CN)6, 5 mM K4Fe (CN)6, 2 mM MgCl2, 0.02% IGEPAL, 0.01% sodium deoxycholate, and 1 mg/mL X-gal in 1×PBS), at 37 °C overnight. On the next day, the embryos were rinsed with PBS. Hence, the wild-type embryos were used as representative negative controls.

### Immunohistochemistry for β-galactosidase detection

Immunohistochemistry was performed according to a previously established protocol 16. Briefly neutral formalin-fixed and paraffin-embedded tissues from heterozygous mice were sectioned at 4 µm in thickness using a microtome (Leica RM2245). Consequently, the rehydrated tissues were then autoclaved in an antigen retrieval 1× solution (pH 6.0, DAKO, Carpinteria, CA, USA) at 121 °C with high pressure for 30 min and then cooled for 1 h without releasing the pressure. After serial washing with distilled water and PBS, these tissues were incubated with antigen blocking solution (DAKO, Carpinteria, CA, USA) at room temperature for 15 min. The tissues were then incubated with a primary antibody against β-galactosidase (rabbit polyclonal, AbD serotec, Oxford, UK) diluted at 1:700 with antibody diluent solution (DAKO) at 4 °C overnight. After rinsing with 1× PBS 3 times (5 min each), the tissues were incubated with polymer-HRP anti-rabbit-labeled secondary antibody (DAKO) for 40 min. Next, a chromogen (DAKO) was used to reveal positive signals. Finally, hematoxylin (ScyTek, Logan, UT, USA) was used for the process of counterstaining in this case.

### Statistical analysis

Data in this study are presented as means and standard deviations. All statistical differences between the groups reviewed were determined by the use of a Student's t-test using a statistical program STATISTICA ver. 8.0 (Tulas, OK, USA). For Mendelian genotype ratios of pups obtained from heterozygote mating, Chi-square test or Fisher's exact test was performed using excel with the real statistics using excel add-in (http://www.real-statistics.com/). In this study the statistical significance was considered at P<0.05.

## Results

### Generation of Ipo11 gene-trap null knock-out mouse

**Figure 1:**
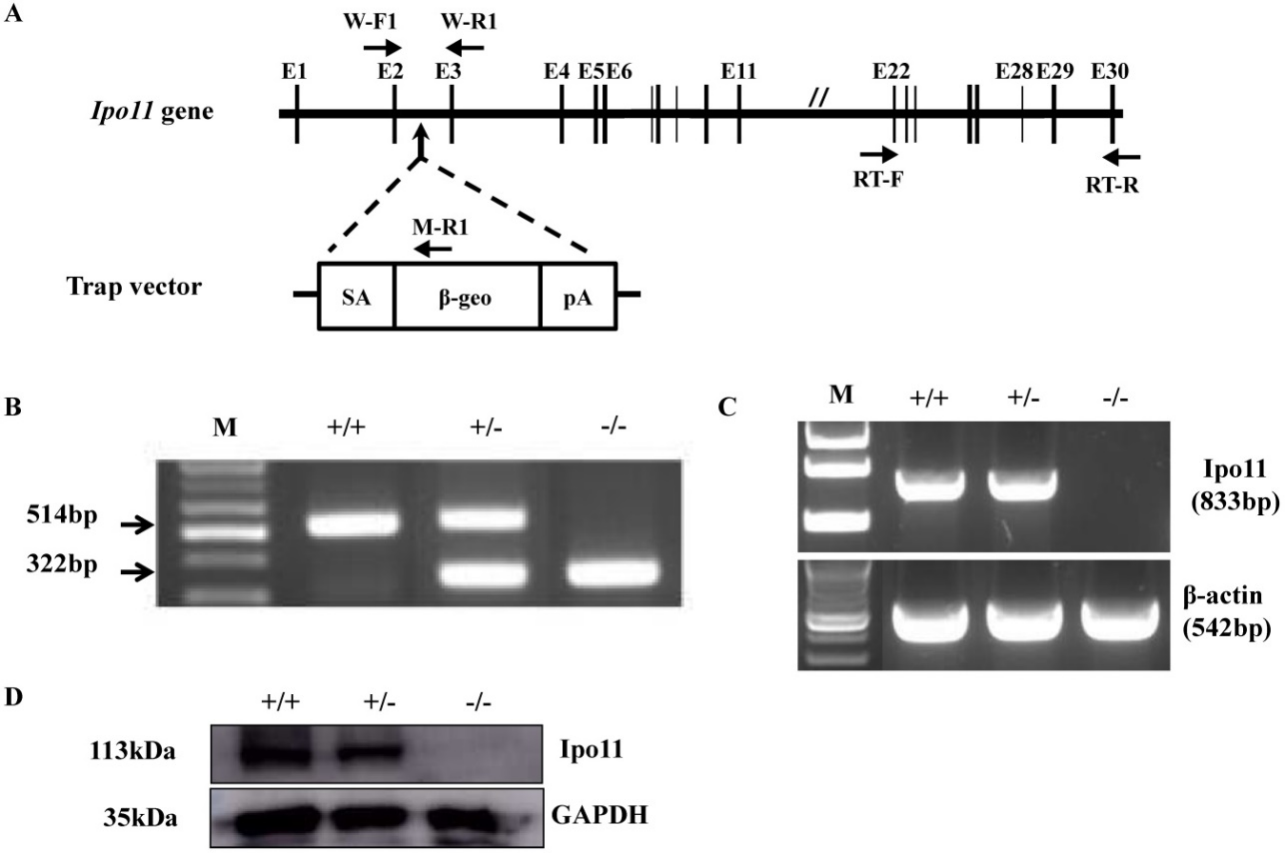

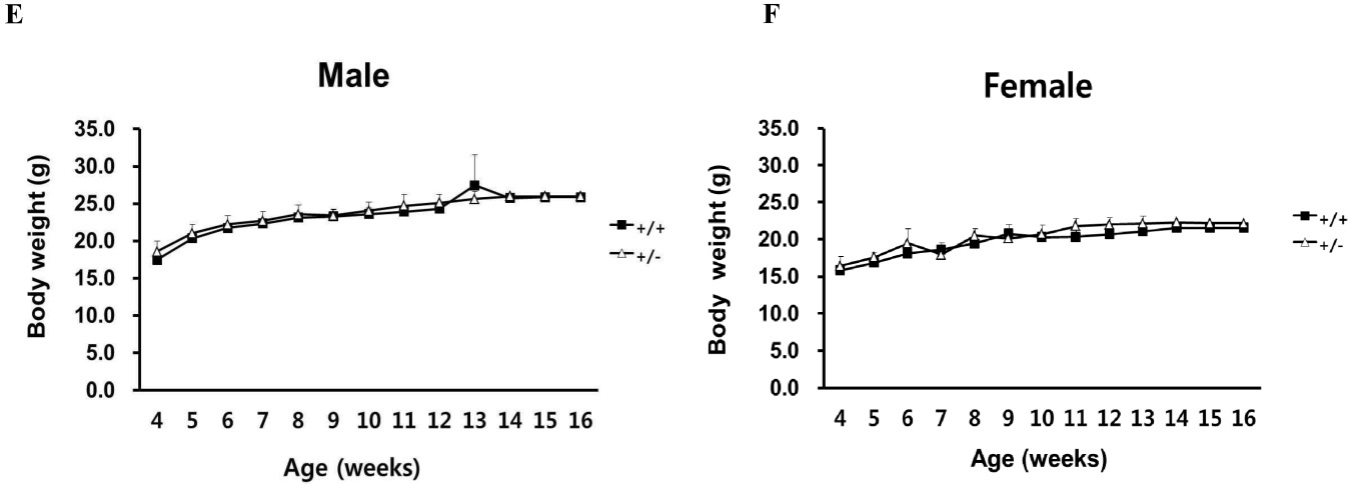
** Generation of Ipo11 mutant mice. (A)** This figure represents the schematic representation of the genomic organization of the murine Ipo11 gene and the insertional mutation resulting from gene-trapping with the pU-21T vector. The locations of the pU-21T vector was inserted gene between exon 2 and exon 3 of the Ipo11 gene comprising 30 exons. The E, exon; SA, splicing acceptor; β-geo, beta-galactosidase and neomycin; pA, polyadenylation site. **(B)** The PCR genotyping analysis to distinguish wild-type allele (514bp) and mutant allele (322bp). PCR primers presented W-F1, W-R1 and M-R1 in Fig 1A. **(C)** Reverse-transcriptase PCR analysis of whole Ipo11^+/+,^ Ipo11^+/-^ and Ipo11^-/-^ E10.5 embryos from exons 22 to 30 clearly demonstrates the loss of the homozygous mutant samples. The RT-PCR primers presented RT-F and RT-R. The PCR reactions were multiplexed with β-actin (lower band) to assess quality of the RNA and cDNA samples. **(D)** Western blotting from whole E10.5 embryo lysates with genotypes indicated above each lane. The filter was developed with anti-Ipo11 and anti-GAPDH antibody as loading control. +/+, +/-, and -/- indicate Ipo11^+/+,^ Ipo11^+/-^, and Ipo11^-/-^ genotypes, respectively. The body weights for male **(E)** and female **(F)** *Ipo11* heterozygous mice were measured every week from 4 to 16 weeks of age. n=8 for each group.

For the generation of *Ipo11* knockout mice (*Ipo11^-/-^*), an ES cell clone mutated by insertion of pU-21T gene trap vector was utilized in this case. The vector insertion site was confirmed to be on the 2nd intron of *Ipo11* gene (Fig. 1A). The use of a PCR analysis using selected primer pairs with genomic DNA could be able to distinguish between the characteristic mutant and wild type alleles (Fig. 1B). As shown in Fig. 1B the three primers could be used to identify the exact genotypes; wild-type allele produced 514 bp-band and mutant allele produced 322 bp-band size.

To confirm the absence of full-length transcripts of Ipo11 gene in the homozygous mutant mouse, RT-PCR analysis using total RNA from E10.5 whole embryos were performed with a primer pair amplifying between exon 22 and exon 30 of *Ipo11* gene. β-actin was used as a control in this case. As a result, a specific PCR band was confirmed corresponding to a size of 833 bp only from the *Ipo11^+/+^*and *Ipo11^+/-^*embryos, but not from *Ipo11^-/-^*embryos. This RT-PCR analysis indicated that the wild-type *Ipo11* transcript is absent in the identified homozygote embryos (Fig. 1C).

In addition, we also performed a western blot analysis with the protein extracts from E10.5 embryos to confirm the loss of Ipo11 protein in homozygote mutants (Fig. 1D) and we confirmed no signal for the homozygous mutant mouse, although a single band was clearly observed for wild type or heterozygous embryos with the expected molecular weight of 113 kDa. Therefore, it was confirmed that the protein level of Ipo11 in the heterozygous embryo was reduced to approximately a half of that in the wild type embryos (upper panel). In this case, GAPDH was used as a loading control (lower panel) (Fig. 1D). These findings indicate that our gene trapping approach induced a complete null mutation in Ipo11 gene in the mouse.

### Survival and growth of Ipo11^-/-^ mice

Upon review and analysis of evaluations in the breeding process, we could not find any homozygous mice among the offspring derived from intercrosses of *Ipo11^+/-^* mice. Therefore it is noted that in this case, eighty-two out of 236 pups were characterized as a wild-type, whereas the remaining 154 were all heterozygotes (Table 1). The ratio between wild and heterozygous offspring was approximately 1:2, suggesting that homozygosity for the *Ipo11* results in embryonic lethality. And there was no difference in the characteristic growth curve between *Ipo11^+/+^* and *Ipo11^+/-^* mice (Fig. 1E and 1F). The absence of homozygous mutant offspring drove us to examine when the embryonic death occur in *Ipo11^-/-^* embryos. The embryos derived from heterozygous intercrosses were collected and genotyped on E18.5, E15.5, E12.5, E11.5, E10.5, E9.5 and E8.5 (Table 1). The genotyping results with embryos from E9.5 or E10.5 revealed a normal Mendelian ratio for *Ipo11* gene, although it is recognized that some embryos undergoing resorption could not be genotyped (Table 1). However, all the homozygous embryos died by E11.5. These results indicate that most *Ipo11*-deficient mice die between the stages of E10.5 embryo and E11.5 embryo.

### Morphological changes of Ipo11^-/-^ mouse embryos

**Figure 2:**
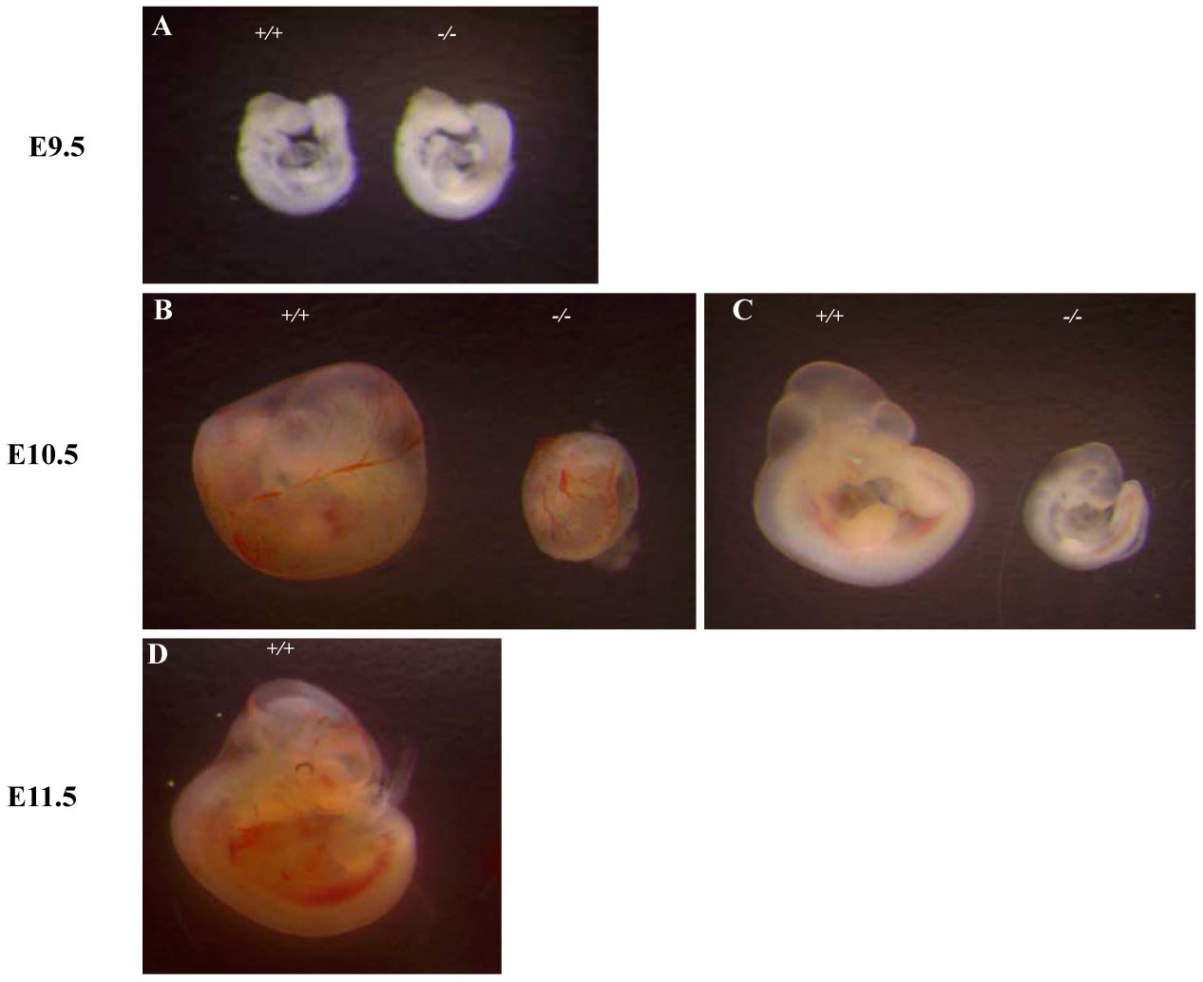
** Abnormal growth and embryonic lethality of *Ipo11^-/-^ mice*.** This represents the whole mount embryos at different stages of development, E 9.5, E 10.5 and E 11.5, as derived from intercrosses of *Ipo11^+/-^*mice. **(A-D)** Bright-field images of *Ipo11^+/+^*(left) and *Ipo11^-/-^*(right) embryos; embryos at E 10.5 embryos within yolk sacs **(B)**, E10.5 embryos with yolk sacs removed** (C)**, and E11.5 embryos **(D)**, respectively.

To investigate the basis for the lethality *of Ipo11^-/-^*, we carried out more detailed analyses of E10.5 embryos (Fig. 2). At E9.5, homozygous embryos could not be distinguished morphologically from their wild-type or heterozygous littermates (Fig. 2A). However, the yolk sacs of *Ipo11^-/-^* embryos was shown to be paler as compared to those of *Ipo11^+/+^* and *Ipo11^+/-^* littermates and their body sizes were much smaller than those of *Ipo11^+/+^* and *Ipo11^+/-^* littermates (Fig. 2B and C), comparable to those of *Ipo11^+/+^* embryos at E9.5 (Fig. 2A). These data suggested that *Ipo11^-/-^* embryos are subject to be abrupt morphological changes during mouse embryonic development at around E10.5 and then they died.

### Expression of Ipo11 gene in embryo and adult mouse

**Figure 3:**
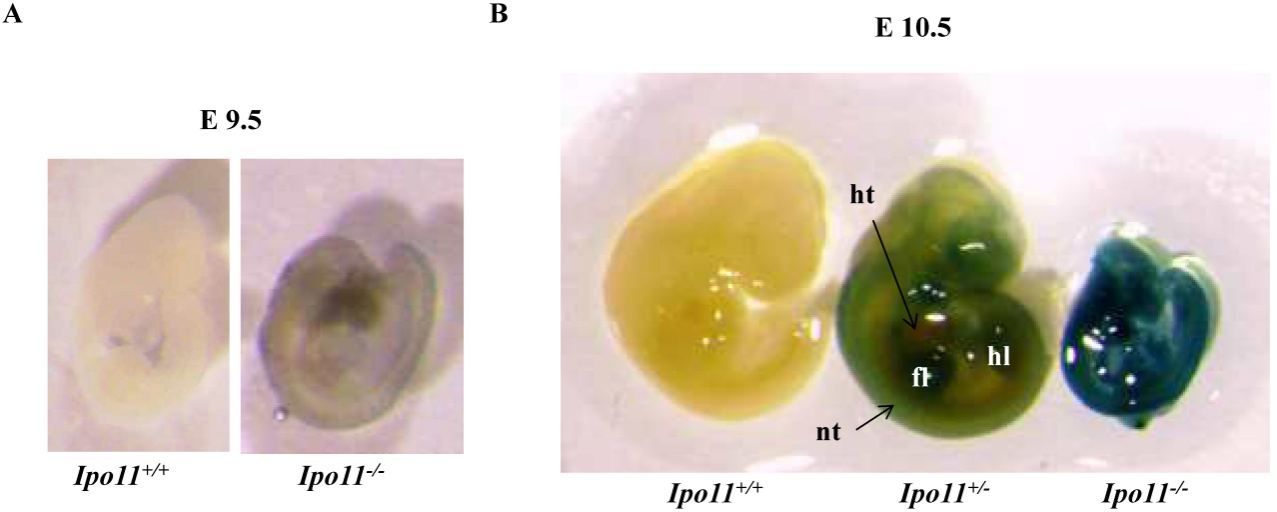
** Expression of Ipo11 during mouse development**. This represents the whole mount lateral views (left panels) and sections (right panels) of Ipo11 embryos at E9.5 **(A)** and E10.5 **(B)** stained with X-gal. *Ipo11-β-gal* expression is specific for the homozygotes and the wild-type embryos do not show any staining for β-galactosidase. The genotypes of the WT and homozygotes were determined using the yolk sacs of the embryos. ht, heart; nt, neural tube; fl, forelimb; hl, hindlimb.

To see whether the embryonic lethality observed in *Ipo11^-/-^* embryos is associated with the gene expression in the embryos, we took advantage of β-galactosidase (lacZ) reporter gene expression under the regulation of the endogenous promoter. Embryos at E9.5 and E10.5 were X-gal stained (Fig. 3). There was only a low level of positive staining signals in the *Ipo11^-/-^* embryos at E9.5 (Fig. 3A). However, *Ipo11^-/-^* embryos at E10.5 showed remarkable staining signals (Fig. 3B). After this stage, the resulting staining rapidly decreased in this case (data now shown).

**Figure 4:**
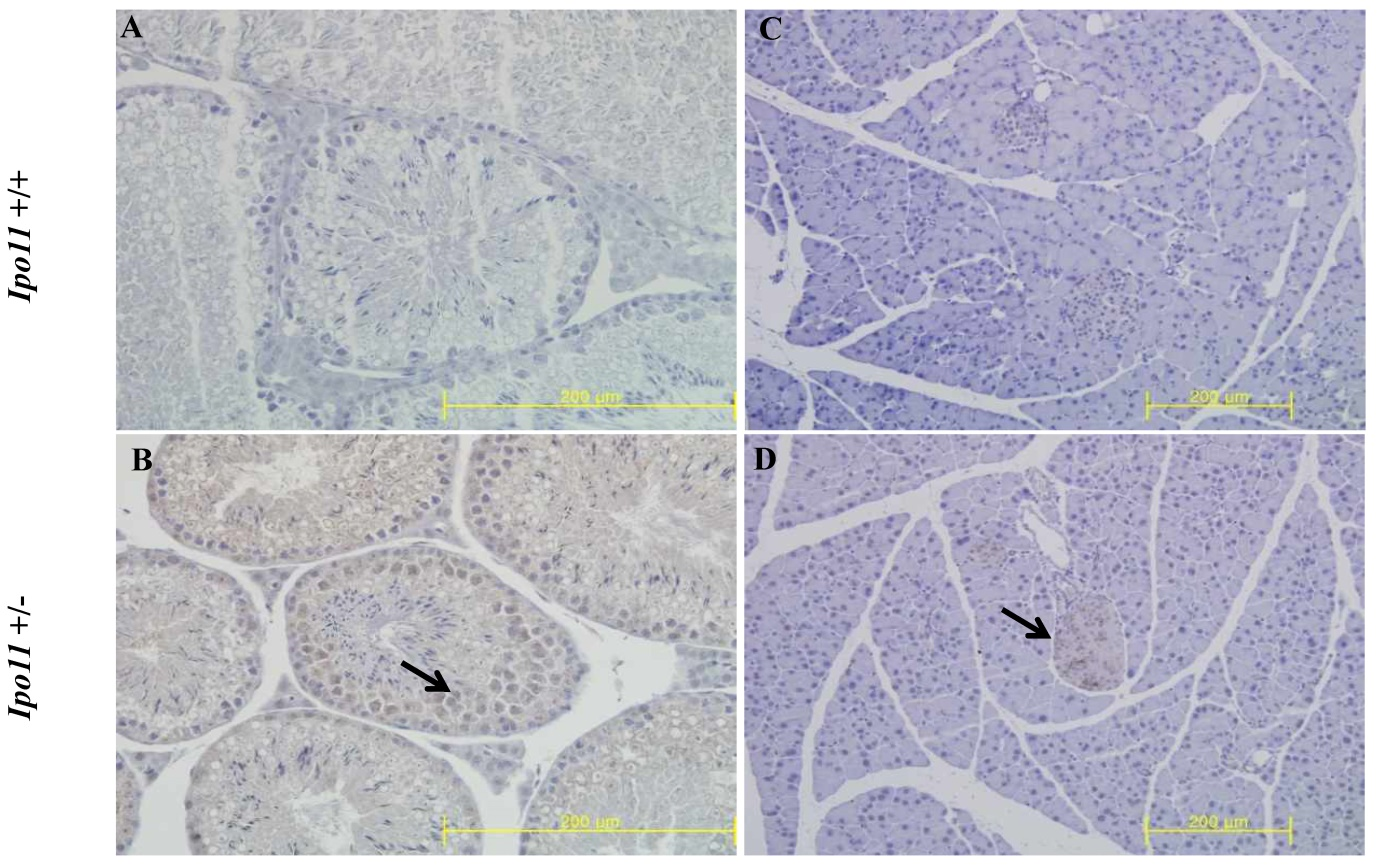
** Immunohistochemical staining of *Ipo11* gene in the testis and pancreas.** Tissues from *Ipo11* heterozygous (+/-) adult mice aged 8 weeks were screened for their *Ipo11* gene expressions by the use of the process of immunohistochemical staining with anti-β-galactosidase antibody. In this case, age matched wild-type (+/+) mice were used as a negative control. In the figure, **(A and B)**, represent the Testis, and **(C and D)**, represent the Pancreas. The size bars represent 200 µm.

We also attempted to find *Ipo11* gene expressing tissues in adult mouse. An analysis of tissues from male and female *Ipo11^+/-^* mice aged 8 weeks were screened for their *Ipo11* gene expressions, by the process of an immunohistochemical staining with anti-β-galactosidase antibody. Among the tissues screened it is noted that the pancreas and the testis were the only tissues including the positive staining signals (Fig. 4). Spermatogonia cells and beta cells were the positive in the tissues, respectively. These results indicated that strong expression of *Ipo11* gene expression is started between E9.5 and E10.5, which is associated with the embryonic death of *Ipo11^-/-^* embryos, and that Ipo11 was expressed in the restricted adult tissues.

## Discussion

A well-known role of transport factors of the importin *β* family is to facilitate the transport of macromolecules that consist of nuclear import or export signals between nucleus and cytoplasm 17,18. To this end, all transport factors are known to constantly shuttle between the nucleus and the cytoplasm. All members have the ability to identify and bind to specific cargoes, either directly or via adaptor molecules, to bind RanGTP and to interact with nucleoporins at the NPC. These characteristic interactions between nucleoporin repeats and importin β family of proteins have been reported both *in vitro* 19-22 and *in vivo* 23 via central transporter of the NPC. These interactions are also important for the import or export of importin β family protein members and their corresponding cargoes via the central transporter of the NPC.

These previous studies indicate that importin-11 mainly involved in the transport of proteins. However, there was no specific research exhibiting its role in developmental processes. In this study, we focused on the roles of importin-11 *in vivo* and in embryogenesis in mouse organism.

We reported the gene trap disruption of a mouse Ipo11 gene and the phenotypic characterization of this mouse model and studied its novel role in mouse embryonic development. The homozygous *Ipo11* mutant mice cannot be recovered among postnatal offspring. Subsequently, it is demonstrated that a disruption of *Ipo11* gene in mice leads to embryonic lethality around E10.5. *Ipo11* gene expression was evident in E10.5 embryos as demonstrated by RT-PCR and western blotting (Fig. 1C and D). The *Ipo11^-/-^* embryos additionally revealed to be developmentally delayed at E10.5 and resulted in death by E11.5 (Fig. 2 and Table 1). *Ipo11^+/-^* mice can survive and show no phenotypic abnormality (data not shown).

To identify Ipo11 expression in adult mice, the lacZ expressions in adult organs were analyzed by immunohistochemistry (IHC) staining with the β-galactosidase antibody. Because the gene-trap vector was consisted of *LacZ* reporter gene, lacZ expressions indicated the Ipo11 gene expressions. The *Ipo11* gene expressions were observed only in pancreas and testis (Fig. 4), as previously demonstrated by DNA microarray data in mouse 24. This immunohistochemical analysis confirmed the expression of *Ipo11* gene in tissues such as the pancreas and testis.

In previous studies, it has been demonstrated that there was a continuous need for the precise transport of large molecules in and out of the nucleus by importin-β proteins (Imp-βs) during the cell cycle and development. Therefore, Ipo 11 might be needed more during embryonic development than during the adult period, and the regulation of Imp-βs expression could be an important factor for mouse development 25-27. DNA-microarray and RT-PCR analysis showed that Ipo11 was expressed during the developmental stages from the fertilized egg to blastocysts, and then until E10.5 in a mouse. In contrast, Ipo11 in human was expressed in the senescent diploid fibroblasts 24. Moreover, the Jevtic et al 13 demonstrated that altering the levels of nuclear import factors in early *Xenopus laevis* embryos affects later development.

Considering the importance of Ipo11 in developmental processes, we need to investigate more about its roles in mouse embryogenesis.

Our present data also suggested that the deficiency of Ipo11 induced embryonic lethality between E10.5 and E11.5 in mice (Table 1). Moreover, our data showed that *Ipo11^-/-^* embryos at E10.5 were much smaller than the *Ipo11^+/+^* or *Ipo11^+/-^* embryos, although it was the same at E9.5 (Fig. 2). In addition, Ipo11 expression was more considerable in E10.5 embryos when compared with that in E9.5 (Fig. 3). Collectively, it is indicated that lack of Ipo11 expression is directly associated with the lethality as observed in the embryos.

Recently, Chen et al have described that Ipo11 is an important nuclear transport receptor for phosphatase and tensin homologue (PTEN), which physically separate PTEN from cytosolic PTEN degradation machinery, indicating *Ipo11* might be a tumor suppressor gene 28. They also showed that low level Ipo11 is closely associated with reduced Pten level in mouse and cancer cell lines. Their hypomorphic (*hy*) *Ipo11* mutant mice expressing reduced mRNA to <25% of normal levels, showed partial embryonic lethality and a strong bias against the reduced protein expression levels 28. In addition, hypomorphic *Pten* mutant mice (*Pten^hy^*) also results in partial embryonic lethality 29, and complete Pten deletion caused embryonic lethality 30. Thus, it is possible that embryonic lethality observed in* Ipo11^-/-^* embryos in this study might be the outcome of a lowered Pten level associated with Ipo11 removal, although we did not measured the Pten level in the mice. We also could not exclude that there may be other critical cargo for Ipo11 uncovered yet.

During the preparation of the manuscript, a report showing that Ipo11 mediates βcatenin nuclear import in some colorectal cancers has been published, indicating that Ipo11 is required for Wnt/βcatenin activation and that Ipo11 might belong to a group of oncogenes 31. As a result, Ipo11 must have diverse roles even in opposite according to the context on the cell death and survival, and there are a lot to be revealed for understanding the full scope of Ipo11 activity.

On the other hand, we could not see any evidences for the mutant allele associated lethality in *Ipo11^+/-^* mice (Table 1), although *Ipo11^-/-^* mutant allele did not expressed any recognizable level of Ipo11 protein. Whereas *Ipo11* hy mutant allele associated lethality were evident in *Ipo11^hy/+^* mice 28. At present, the reason for this lethality difference associated with gene expression level is not clear. However, the site differences of the mutations introduced on the *Ipo11* gene might be related with that, intron 5 for *Ipo11* hy allele and intron 2 for *Ipo11^-/-^* mutant allele in this study.

These observations led us to conclude that regulation of Ipo11expression could be an important factor for mouse embryonic development and deletion of Ipo11 nuclear import factor affects normal embryonic development, and it may be seen as a variable which governs embryonic lethal phenotypes in a mouse. Further analysis of the detailed action mechanism of Ipo11 will be required for better understanding embryonic lethality governed by lack of *Ipo11* gene in mice.

## Figures and Tables

**Table 1:** Genotype analysis of *Ipo11^+/-^* intercross progeny

Stage	Genotype			ND	p-value*	Total
	+/+	+/-	-/-			
4 weeks	82	154	0	-	0.000	236
E18.5	2	6	0	0	0.504	8
E15.5	3	10	0	2	0.187	16
E12.5	2	8	0	4	0.406	14
E11.5	7	16	0	5	0.037	28
E10.5	18	28	16	4	0.872	66
E9.5	6	13	5	2	1.000	26
E8.5	2	5	4	0	0.880	11

E, day of the embryonic development. ND, not determined. *, Chi-square test or Fisher's exact test were performed for goodness of fit to the expected Mendelian ratios 1:2:1.
